# Low social status decreases the neural salience of unfairness

**DOI:** 10.3389/fnbeh.2014.00402

**Published:** 2014-11-20

**Authors:** Jie Hu, Yuan Cao, Philip R. Blue, Xiaolin Zhou

**Affiliations:** ^1^Center for Brain and Cognitive Sciences and Department of Psychology, Peking UniversityBeijing, China; ^2^Key Laboratory of Machine Perception (Ministry of Education), Peking UniversityBeijing, China; ^3^PKU-IDG/McGovern Institute for Brain Research, Peking UniversityBeijing, China

**Keywords:** social status, social hierarchy, fairness, ultimatum game, ERP, P2, N400, LPP

## Abstract

Social hierarchy exists in almost all social species and affects everything from resource allocation to the development of intelligence. Previous studies showed that status within a social hierarchy influences the perceived fairness of income allocation. However, the effect of one’s social status on economic decisions is far from clear, as are the neural processes underlying these decisions. In this study, we dynamically manipulated participants’ social status and analyzed their behavior as recipients in the ultimatum game (UG), during which event-related potentials (ERPs) were recorded. Behavioral results showed that acceptance rates for offers increased with the fairness level of offers. Importantly, participants were less likely to accept unfair offers when they were endowed with high status than with low status. In addition, cues indicating low status elicited a more positive P2 than cues indicating high status in an earlier time window (170–240 ms), and cues indicating high status elicited a more negative N400 than cues indicating low status in a later time window (350–520 ms). During the actual reception of offers, the late positivity potential (LPP, 400–700 ms) for unfair offers was more positive in the high status condition than in the low status condition, suggesting a decreased arousal for unfair offers during low status. These findings suggest a strong role of social status in modulating individual behavioral and neural responses to fairness.

## Introduction

Fairness is an essential social norm in interpersonal interaction (Fehr and Fischbacher, 2003). A wide variety of economic games shows that people demand fairness in wealth allocation and are often willing to sacrifice their own interest to punish behaviors that they feel are unfair (Fehr and Fischbacher, 2003; Corradi-Dell’Acqua et al., 2013; Pedersen et al., 2013). In one such game, the ultimatum game (UG), one player acts as the proposer and is given a set amount of money to allocate to the recipient who, in turn, can either accept the offer, resulting in the allocation designated by the proposer, or reject the offer, resulting in both parties coming away with nothing (Güth et al., 1982). According to traditional economic theory, people are motivated by self-interest, which means that proposers should offer the lowest acceptable amount and recipients should accept any non-zero offer. Yet research shows that proposers tend to split the pot evenly; recipients will reject unfair offers, and the rejection rate increases as a function of the level of unfairness. Such “irrational” decisions, some researchers have argued, reflect norm-based preferences for equality (Camerer and Fehr, 2006) or personal reputation (Sanfey et al., 2003).

Fairness considerations are influenced by a number of social factors such as goodwill intention (Handgraaf et al., 2003; Güroğlu et al., 2010), initial ownership (Wu et al., 2012), personal reputation (Charness et al., 2010), and social comparison (Wu et al., 2011b). One other critical factor is the social status of the individuals involved. Social status, or social rank, refers to an individual’s relative position in terms of wealth, ability, education, stature, or profession in a hierarchy (Adler et al., 2000; Zink et al., 2008; Kraus et al., 2011b). As a highly pervasive principle of social organization across almost all species (Chiao, 2010), social hierarchy affects the way we see ourselves and others (Zink et al., 2008) and influences both physical health (Sapolsky, 2005) and intellectual development (Bates et al., 2013). Individuals in high standing often have preferential access to resources vital to survival, including food, land, information, power, and potential mating partners; they also have more power or influence over individuals in lower standing, making inference of others’ status for such individuals extremely important in social interactions (Chiao et al., 2004). Status can be inferred from interpersonal features such as facial expression (Chiao et al., 2008), body posture (Marsh et al., 2009), performance hierarchy (Zink et al., 2008; Breton et al., 2014), and from social signals including military symbols (Chiao et al., 2009).

Of interest to the present study is that social status may influence the way we engage in wealth allocation. It has been found that individuals in low social standing are more generous, charitable, trustworthy, and helpful than their high status counterparts, who are more likely to break laws (e.g., fail to break for a pedestrian while driving) and social norms (e.g., take candy from a child, report false scores to their advantage) (Piff et al., 2010, 2012). Low status individuals also charge less than high status individuals in bargaining situations (Ball et al., 2001). In one study, Albrecht et al. (2013) used performance on a quiz to establish participants’ social status and then instructed them to judge how satisfied they would be if given certain offers (ranging from disadvantageous unfair to advantageous unfair). Individuals endowed with inferior social status were more satisfied with disadvantageous payoff inequalities than their higher status counterparts.

However, social status in these studies was assigned either randomly or based on participants’ performance in a particular task. Throughout each study, social status was fixed for a particular participant and the comparison for the effect of social status on social or economic decisions was between participants, limiting the generalizability of the results. For instance, in all of the above-mentioned studies, social status was either measured by objective factors (i.e., socioeconomic status) or manipulated at one point in time (priming, Piff et al., 2012; random assignment, Ball et al., 2001; performance on a trivia quiz, Ball et al., 2001; Albrecht et al., 2013), making it unclear whether the effects of social status would change as social status changes. Additionally, Albrecht et al. measured the effects of relative status (lower, same, higher status) on potential satisfaction with a wide array of hypothetical offers. However, this does not provide insight related to fairness perception of offers made in a real social interaction and eliminates the potential for a graded examination of status on offer satisfaction. Finally, these studies focused on the behavioral effects of social hierarchy, without accounting for the neural substrates of these effects.

**Figure 1:**
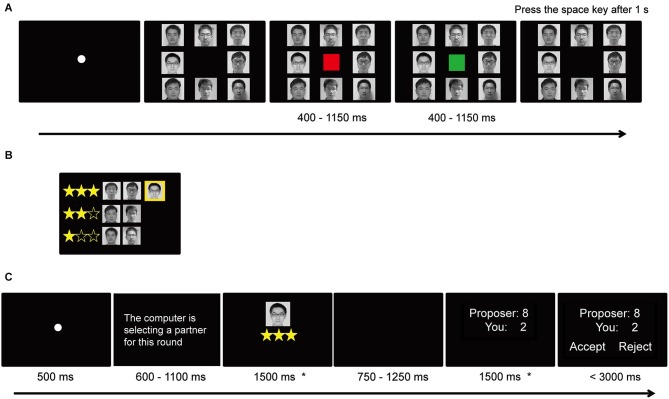
**Schematic diagram of the experiment**. Each block in the experiment consisted of two sessions: the status-inducing session and the UG session. In the status-inducing session, the EEG participant completed 5 rounds of the time estimation task together with 7 other players by attempting to press the space key exactly 1 s after the green square disappeared **(A)**, and then viewed status information based on his/her estimation accuracy relative to others **(B)**. The EEG participant’s photo was highlighted with a yellow background. In UG session **(C)**, the participant saw the cues indicating his/her social status before the reception of UG offers. We focused on the ERP responses time-locked to the screens with the asterisk.

In the current study, we used an interactive rank-inducing task (i.e., time estimation task; see Boksem et al., 2012) to dynamically manipulate participants’ social status over time and then asked participants to act as recipients in UG. In the rank-inducing task, a participant estimated the passing of 1 second together with 7 partners (Figure 1A). Then he or she was given a rank (high, medium, or low) according to his or her estimation accuracy relative to the accuracy of the other players (Figure 1B). Following the ranking, the participant played UG with one proposer randomly drawn from the partners. During each UG trial, the participant was first presented with a cue indicating his/her social status (high, medium, or low) acquired in the previous rank-inducing task and then presented with the UG offer ostensibly given by the proposer (Figure 1C). The reason for presenting the social status cue was twofold: first, this cue served to reinforce the effects of the rank-inducing task throughout the UG trials; second, by measuring this cue on every trial, we were able to measure the neural processing of one’s own social status, independently of offers in UG. After several rounds of UG, the participant entered the next block of the rank-inducing task during which he or she would attain a new rank before entering the next rounds of UG. The fairness of the offer in UG was varied systematically over different rounds of the game. We recorded event-related potentials (ERPs) time-locked to the cue indicating participants’ social rank and to the offers in UG. The empirical question was how the brain responded to the social status cues and how social status modulated the subsequent acceptance rate and neural responses to the offers in UG.

Behaviorally, we focused on the acceptance rate of UG offers. In line with previous studies, we expected that the acceptance rate would decrease as the level of fairness in the division scheme decreases. Importantly, we predicted that when participants occupied low status, compared with occupying high status, they would feel less entitled to fair offers and, as a result, be more likely to accept unfair offers. This prediction is supported by studies showing that low status individuals charge less than high status others when bargaining (Ball et al., 2001) and are more satisfied with unfair offers than when endowed with high status (Albrecht et al., 2013). This “entitlement hypothesis” contrasts with an alternative “emotion hypothesis”, which assumes that participants occupying low status may have increased negative emotions towards unfair offers and are therefore *less* likely to accept them as a result. When facing threats from others, individuals experience stronger negative emotions when they are in a low status position than in a high status position (Kraus et al., 2011a). In fact, when given the opportunity to express emotions in UG, acceptance rates increase, suggesting that rejection of unfair offers may be an expression of negative emotion (Xiao and Houser, 2005). Combined with previous research showing that negative emotional states decrease the likelihood of offer acceptance regardless of offer amounts in UG (Harlé and Sanfey, 2007), one might predict that participants in low status will accept *less* unfair offers than while in high status.

At the neural level, we predicted that brain responses to the cues indicating the status information should exhibit an attentional effect with increased P2 or P3/LPP responses for the low status when compared with the high status. This prediction is tentative due to the lack of research on the neural temporal processing of social status information. Despite this gap, there is an abundance of potentially relevant research on the neural processing of emotions (Olofsson et al., 2008). Emotions are relevant when it comes to social status because different ranks lead to different levels of emotion (Zink et al., 2008). We expected that, compared with the cue indicating high status, the cue indicating low status would evoke more negative emotions which would increase attention to the cue. Negative emotional stimuli elicit an increased P2 when compared with positive emotional stimuli (Carretié et al., 2004; Delplanque et al., 2004); positive emotional stimuli also elicit an increased P3 or late positivity potential (LPP) when compared with neutral stimuli (Bublatzky et al., 2014). In addition, given that the social status cues were composed of the participant’s own face and star ranking information (Figure 1C), these cues would also be expected to invoke a self-evaluation process linking the cue with self-related knowledge representations. Previous studies have shown that, relative to unfamiliar faces, self faces or familiar faces elicit a more negative N400 (Bentin and Deouell, 2000; Eimer, 2000; Caharel et al., 2006; Butler et al., 2013). This N400 effect reflects the process of integrating one’s own face with self-related representations in long-term memory (Guillem et al., 2001; Caharel et al., 2006), possibly for the purpose of self-enhancement (Brown et al., 1988). We may therefore predict more negative N400 responses to one’s own face associated with high status relative to the same face associated with low status.

For the neural responses to the offers in UG, we predicted that, compared with fair offers, unfair offers would elicit an enhanced medial frontal negativity (MFN, or feedback-related negativity, FRN) and a decreased P300. The MFN or FRN, which is a negative deflection peaking between 200 ms and 350 ms post-onset of feedback at frontocentral electrodes, has usually been shown to be more enhanced for unfair offers than for fair offers (Polezzi et al., 2008; Boksem and De Cremer, 2010; Hewig et al., 2011; Wu et al., 2012). It is suggested that the MFN or FRN reflects an earlier, automatic detection of social expectancy violation (Wu et al., 2011a). The P300, which is the most positive peak in the period of 200–500 ms post-onset of feedback at frontoparietal electrodes, has been found to be smaller for unfair offers than for fair offers (Wu et al., 2011b; Qu et al., 2013). It is suggested that the P300 reflects later, high-level motivational/emotional processes (Yeung and Sanfey, 2004). In one study, unfair offers were also found to elicit a smaller LPP than fair offers in a relatively late time window (450–650 ms) (Wu et al., 2011b), which is perhaps not surprising given that LPP and P300 are commonly assumed to originate from the same sources (Hajcak et al., 2010). Critically, we were interested in how social status modulates the response of the P300/LPP to offer fairness. Although social factors, such as social comparison (Wu et al., 2011b) and social exclusion (Qu et al., 2013), do not affect MFN, they do affect the later ERP components. Thus, it is highly likely that the social status would influence the P300/LPP effects on offer fairness.

## Methods

### Participants

Thirty-two undergraduate and graduate students participated in the experiment. Six of them were excluded for various reasons (see the Result section). The remaining twenty-six participants (12 females) aged between 18 and 25 years (mean age 22.3 years, SD = 2.1). All the participants were healthy, right-handed, and had normal or corrected-to-normal vision. No participant had a history of neurological or psychiatric disorders. Each participant was informed that the basic payment would be around 80 Chinese yuan (about 13.5 USD) and additional monetary reward would fluctuate based on performance in the experiment (in actuality, all participants received 100 Chinese yuan, about 16.5 USD). Informed consent was obtained from each participant before the experiment. The experiment was in accordance with the Declaration of Helsinki and was approved by the Ethics Committee of the Department of Psychology, Peking University.

### Design and procedure

The experiment had a 2 × 3 within-participant factorial design, with the first factor referring to social status (low vs. high) and the second factor referring to offer fairness (unfair vs. sub-fair vs. fair). As the focus of the study was on the difference between high and low status, we set the medium status rank as a filler condition. The UG offer was operationally defined unfair if it was under 3 out of 10 yuan, sub-fair if it was between 3 and 4 out of 10 yuan, and fair if it was higher than 4 out of 10 yuan. No offer was greater than 5 out of 10 yuan. We defined a sub-fair level of offers because recipients require more complex computations or strategies to decide whether to accept or reject such offers than to respond to fair and unfair offers (Polezzi et al., 2008). The social status information was conveyed through a set of stars, with one filled star and two empty stars indicating the low rank and three filled stars indicating the high rank (two filled stars and one empty star indicated the middle rank).

Upon arriving at the laboratory, the participant briefly met a same-sex confederate and was told that the two of them were going to act as recipients in UG in separate EEG rooms, which would involve six same-sex strangers acting as proposers; the six proposers would ostensibly arrive 1 h later because they did not need to prepare for EEG recording. As such, the participant did not meet the six proposers face to face and only viewed pictures of their faces during the experiment. The reason for such a setting was threefold: to form dynamic social hierarchies across blocks, to avoid a reputation building effect in the ultimate game, and to increase the interactivity and credibility of the experiment.

The experiment consisted of two alternating tasks (Figure 1). The first was a time estimation task (Boksem et al., 2012). At the start of each time estimation task trial, the participant viewed the facial photos (grayscale) of all eight players on the screen, which indicated that the eight players were simultaneously performing the task. Then, a red square appeared at the center of the screen as a warning signal and turned green after 400–1150 ms. The green square disappeared after 400–1150 ms; the participants were instructed to press the space key on the keyboard exactly 1 s after the offset of the green square. They were told that their relative performance on the task would determine their ranking. After completing 5 trials of this task, all players were ranked and the participant received the ranking information (which was experimentally manipulated; Figure 1B). To avoid any confounding effect of social comparison between the participant and the other recipient (confederate), we only presented the ranking information of the participant and that of the other six persons who would act as proposers in the subsequent UG. The initial rank (high, middle, and low) in the first block was counterbalanced across participants.

The second task was UG. Each trial began with the presentation of a fixation sign (a white cross subtended 0.3° of visual angle) for 500 ms against a black background. The sentence “The computer is selecting a partner for this round” in Chinese (white and Song font, size 32) was presented for either 600, 700, 800, 900, 1000, 1100 ms, informing the EEG participant that one of the other six proposers was randomly being paired with the partner for the current trial. After the partner was successfully chosen by the computer, the EEG participant’s own picture (face only, grayscale, subtended 1.5° × 1.6°) and his/her own rank information (denoted by a set of stars, subtended 2° × 0.8°) were presented at the center of the screen for 1500 ms (Figure 1C). After the presentation of a blank screen for a jittered time between 750 and 1250 ms, the proposer’s division scheme (e.g., “Proposer: 8, You: 2”, white and Song font, size 32) was shown at the center of screen for 1500 ms. After the offer presentation, the two options, “accept” and “reject”, appeared on the left and right side of the screen respectively, with their positions randomly switched over trials. The EEG participant was asked to make the “accept” or “reject” decision by pressing the corresponding key as quickly as possible (using the index fingers of the left and right hands). The next trial began 1000 ms after the key press. Both the acceptance rate and the response latency were recorded. The participant was reminded that the proposers made their decisions individually and independently, and his/her decisions would not be revealed to the proposer.

Throughout the experiment, we recorded EEG data. We focused our analysis on the cue indicating social status and the offer in UG. It is important to note that the cue indicating status was given on every trial during UG and only included the photo and rank of the participant him/herself. We did not evaluate ERPs on the collective game rank screen (Figure 1B) because this ranking screen was only given once per block, resulting in too few data points for reliable analysis.

The participant was seated comfortably about 1.5 m in front of a computer screen in a dimly lit and electromagnetically shielded room. The experiment was administered on a computer with a VisuoSonic 22-in. CRT display, using Presentation software (Neurobehavioral System Inc.) to control the presentation and timing of stimuli. The experiment consisted of 12 blocks with each block including 5 trials of time estimation task (resulting in one rank for the participant) and 30 trials of UG. The participant was endowed with each of the two critical levels of rank—high and low—for 4 blocks, with another 4 blocks of middle rank as filler. To reduce the practice and fatigue effect, the sequence of rank was Latin-squared across participants.

There were 10 trials for each of the offer levels (unfair: 1/9, 1.5/8.5, 2/8, 2.5/7.5; sub-fair 3/7, 3.2/6.8, 3.8/6.2, 4/6; fair: 4.2/5.8, 4.5/5.5, 4.8/5.2, 5/5) for each critical social status condition (high vs. low). The number before the slash denoted the amount offered to the recipient and the number after the slash denoted the amount given to the proposer. Unknown to the participant, all the offers were predetermined by a computer program and pseudo-randomized with the restriction that no more than 3 consecutive trials were of the same offer fairness.

Before the formal test, the participant performed 20 trials of the time estimation task and 10 trials of UG to become familiar with the two tasks. After the experiment, the participant was asked to indicate on a 7-point Likert Scale to what extent he/she perceived his/her status as higher/lower (1 = much higher, 7 = much lower) than other players when he/she was in each status condition. He/she was also asked to indicate his/her minimal acceptable amount (out of 10 yuan) in UG, which was taken as the measure of the perceived entitlement of the asset, and fairness expectation within each social rank. In order to test the effect of social status on emotions, participants reported on a 5-point Likert scale (1 = not at all, 5 = very strongly) the extent to which they felt the intensity of negative and positive emotions during each level of social status. The negative emotion included the following dimensions: irritable, uneasy, nervous, uncomfortable, angry, and shameful, whereas the positive emotion included the following dimensions: interested, energetic, proud, inspired, determined, excited, happy, satisfied, and superior. The participant was paid, debriefed, and thanked at the end of the experiment.

### EEG recording and analysis

EEGs were recorded from 64 scalp sites using tin electrodes mounted in an elastic cap (Brain Products, Munich, Germany) according to the international 10–20 system. The vertical electrooculogram (VEOGs) was recorded supra-orbitally from the right eye. The horizontal EOG (HEOG) was recorded from electrodes placed at the outer cantus of left eye. All EEGs and EOGs were referenced online to an external electrode, which was placed on the tip of nose and were re-referenced offline to the mean of the left and right mastoids. Electrode impedance was kept below 5 kΩ for all the electrodes. The bio-signals were amplified with a band pass from 0.016 to 100 Hz and digitized on-line with a sampling frequency of 500 Hz.

Separate EEG epochs of 1000 ms (with a 200-ms pre-stimulus baseline) were extracted offline, time-locked to the onset of each offer as well as to the onset of the screen in which the participant’s portrait was presented with his/her rank information in UG. Ocular artifacts were corrected by using independent component analysis (ICA) approach. The EEG data were filtered with a band-pass from 0.016 to 30 Hz. Epochs were baseline-corrected by subtracting from each sample the average activity of that channel during the baseline period. All trials in which EEG voltages exceeded a threshold of ± 80 µV during recording were excluded from further analysis. For the cue indicating social status, on average 90% (SD = 8%) of the epochs after artifact rejection were entered into statistical analysis; for UG offers, on average 94% (SD = 5%) of the epochs after artifact rejection were entered into statistical analysis.

For statistical analysis, we divided electrodes into 9 regional clusters based on two three-level factors: Hemisphere (left vs. medial vs. right) and Region (anterior vs. central vs. posterior). The left anterior cluster included F3, F5, FC3 and FC5; the medial anterior cluster included F1, Fz, F2, FC1, FCz and FC2; the right anterior cluster included F4, F6, FC4 and FC6; the left central cluster included C3, C5, CP3 and CP5; the medial central cluster included C1, Cz, C2, CP1, CPz and CP2; the right central cluster included C4, C6, CP4 and CP6; the left posterior cluster included P3, P5 and PO7; the medial posterior cluster included P1, Pz, P2, PO3, POz and PO4; the right posterior cluster included P4, P6 and PO8. Averaged amplitude over electrodes in each regional cluster was used for statistical purposes. Time windows were selected according to visual inspection of the waveforms and preliminary analyses. For ERP responses to different levels of rank, we focused on the P2 (the mean amplitudes in the time window of 170–240 ms) and the N400 (the mean amplitudes in time window of 350–520 ms). Analyses of variance (ANOVAs) were conducted with three within-participant factors: social status (high vs. low), hemisphere (left vs. medial vs. right), and region (anterior vs. central vs. posterior). For ERP responses to different offer levels, we focused on the MFN (the mean amplitudes in the time window of 260–360 ms) and the LPP (the mean amplitudes in the time window of 400–700 ms). ANOVAs were conducted with four within-participant factors: social status (high vs. low), offer fairness (unfair vs. sub-fair vs. fair), hemisphere (left vs. medial vs. right), and region (anterior vs. central vs. posterior). The Bonferroni correction was used for multiple comparisons and the Greenhouse-Geisser correction was applied for nonsphericity when necessary.

## Results

Among the thirty-two EEG participants, three participants claimed after the experiment that they disbelieved the setup of the study, two participants accepted all offers regardless of the fairness level, and one participant showed excessive artifacts in EEG recording. These participants were excluded from data analysis, leaving twenty-six participants for the following analysis.

### Manipulation checks of social status

The post-experiment manipulation check for social status asked participants how inferior/superior they felt in relation to the other players after attaining one (three) stars in the time-estimation game (1 = very inferior; 7 = very superior). Participants’ responses suggested that the star ranking in the time estimation task strongly influenced the perception of social status. A one-factor (star ranking: three vs. one) repeated measures ANOVA on the perceived status showed a significant main effect of star ranking, *F*_(1,25)_ = 147.90, *p* < 0.001, *η^2^_partial_* = 0.86. When the participants obtained three stars in the time estimation task, they perceived themselves as being in a higher status (mean ± SE, 5.27 ± 0.16) than in the one-star, low status condition (2.15 ± 0.16).

The manipulation of social status also affected the participants’ self-reported minimal acceptable amount in UG (out of 10 yuan). The minimum acceptable offer was significantly higher when participants were in high status (3.73 ± 0.20) than in low status (3.28 ± 0.19), *F*_(1,25)_ = 5.57, *p* < 0.05, *η^2^_partial_* = 0.18. Participants’ fairness expectations were higher in high rank status (5.03 ± 0.14) than in low rank status (4.35 ± 0.17), *F*_(1,25)_ = 8.15, *p* < 0.01, *η^2^_partial_* = 0.25. These results suggest that the perceived fairness in asset allocation was modulated by social status.

Finally, social status also affected participants’ experience of emotions. We averaged scores on different dimensions to give overall scores for the negative and positive emotions. ANOVA showed that participants experienced more negative emotion in low status (2.11 ± 0.12) than in high status (1.47 ± 0.08), *F*_(1,25)_ = 25.3, *p* < 0.001, *η^2^_partial_* = 0.50. Additionally, participants experienced less positive emotion during low status (1.77 ± 0.10) than during high status (3.87 ± 0.16), *F*_(1,25)_ = 262.3, *p* < 0.001, *η^2^_partial_* = 0.91.

### Behavior results

We first averaged the response times for unfair, sub-fair, and fair offers over each participant, and performed a one-way repeated-measures ANOVA on the response time of the three types of offers in UG. This analysis revealed a significant main effect of offer fairness, *F*_(2,50)_ = 21.52, *p* < 0.001, *η^2^_partial_* = 0.46, with longer response times for the sub-fair offers (905 ms) than for unfair (844 ms,* p* < 0.001) and fair offers (824 ms,* p* < 0.001). In line with Polezzi et al. (2008), our findings suggest that sub-fair offers elicited a more complex computation or strategy than fair and unfair offers. Therefore, predefining each offer level in a smaller range and distinguishing sub-fair offers from unfair offers can help us show clearer and more precise behavioral and neural responses to each type of offers.

**Figure 2:**
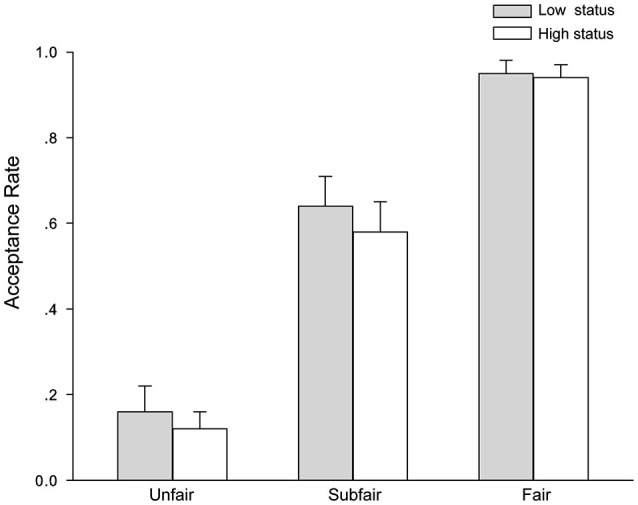
**The acceptance rate depicted as a function of offer fairness and social status**. Error bars represent standard errors of the means.

We then performed a 2 (social status: high vs. low) × 3 (offer fairness: unfair vs. sub-fair vs. fair) repeated-measures ANOVA on participants’ acceptance rates for different offers in UG. This analysis revealed a significant main effect of offer fairness, *F*_(2,50)_ = 107.74, *p* < 0.001, *η^2^_partial_* = 0.81, with the lowest acceptance rate for unfair offers (0.14 ± 0.05), intermediate for sub-fair offers (0.61 ± 0.07), and highest for fair offers (0.95 ± 0.03). The differences between conditions were all significant, *p*s < 0.001. Importantly, the main effect of social status was also significant, *F*_(1,25)_ = 5.21, *p* < 0.05, *η^2^_partial_* = 0.17, indicating that the overall acceptance rate was higher when the participants were endowed with low status (0.59 ± 0.04) than high status (0.55 ± 0.04). There was no significant interaction between social status and offer fairness on acceptance rate *F*_(2,50)_ < 1. Figure 2 shows the acceptance rates for different offers.

### P2 in the 170–240 ms time window on the cue indicating social status

**Figure 3:**
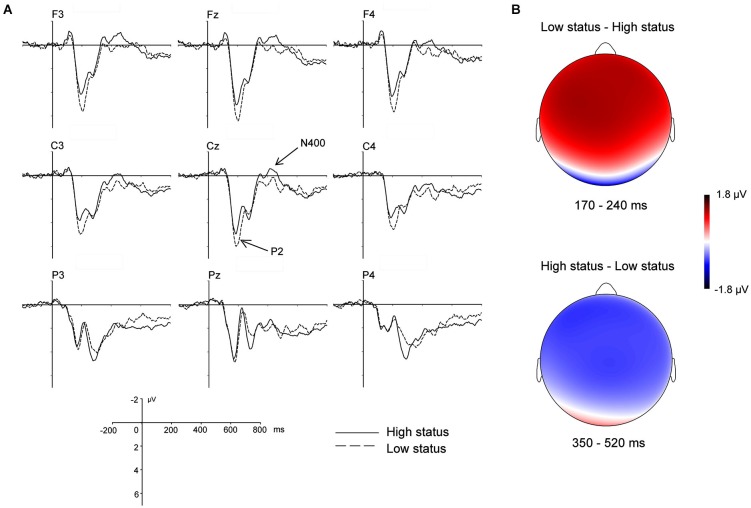
**ERP responses and topographic maps on the cues indicating social status. (A)** ERP responses time-locked to the onset of the social status cue at the exemplar electrodes F3, Fz, F4, C3, Cz, C4, P3, Pz, and P4. The mean amplitudes of the P2 were calculated within the 170–240 ms time window. The mean amplitudes of the N400 were calculated within the 350–520 ms time window. **(B)** Topographic map for the P2 effect in the 170–240 ms time window, upper; topographic map for the N400 effect in the 350–520 ms time window, lower.

We measured ERP responses to the presentation of cues which denoted participants’ social status (Figure 3). In the time window of 170–240 ms, a 2 (social status: high vs. low) × 3 (hemisphere: left vs. medial vs. right) × 3 (region: anterior vs. central vs. posterior) repeated measures ANOVA revealed a significant main effect of social status, *F*_(1,25)_ = 8.38, *p* < 0.01, *η^2^_partial_* = 0.25, indicating that ERP responses to the low status cue (3.23 ± 0.37 µV) were more positive-going than ERP responses to the high status cue (2.62 ± 0.33 µV), *p* < 0.01. The interaction between social status and region was also significant, *F*_(2,50)_ = 40.87, *p* < 0.001, *η^2^_partial_* = 0.62. Testing for simple effects suggested that the P2 effect appeared mostly in the anterior and central regions (*p*s < 0.01), not in the posterior regions (*p*s > 0.1).

### N400 in the 350–520 ms time window on the cue indicating social status

In the time window of 350–520 ms, the 2 × 3 × 3 ANOVA showed a significant main effect of social status, *F*_(1,25)_ = 5.11, *p* < 0.05, *η^2^_partial_* = 0.17, indicating that the high status cue elicited a more negative-going N400 (0.92 ± 0.26 µV) than the low status cue (1.33 ± 0.23 µV). The interaction between social status and region was also significant, *F*_(2,50)_ = 6.34, *p* < 0.05, *η^2^_partial_* = 0.20. Further analysis showed that the N400 effect was in the anterior and central regions (*p*s < 0.05), not in the posterior regions (*p*s > 0.1).

### MFN in the 260–360 ms time window on the presentation of UG offers

**Figure 4:**
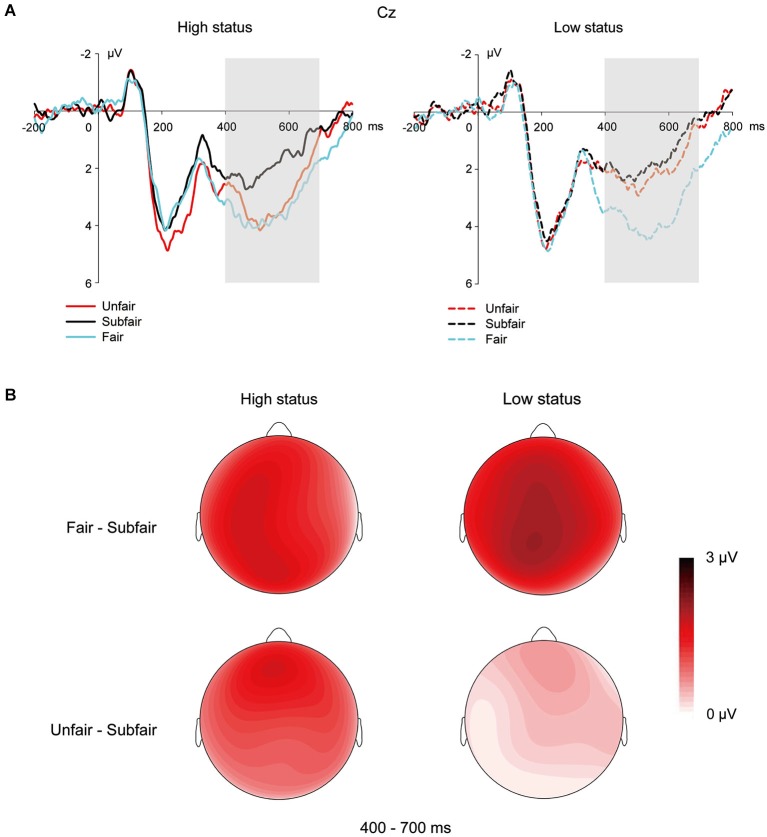
**ERP responses and topographic maps on the UG offers. (A)** ERP responses time-locked to the onset of the offer at the exemplar electrode Cz. The shaded 400–700 ms time window represents the area used for the calculation of LPP mean amplitudes. **(B)** Topographic maps for the LPP effects in the 400–700 ms time window.

For ERPs locked with UG offers (Figure 4), in the time window of 260–360 ms, a 2 (social status: high vs. low) × 3 (offer fairness: unfair vs. sub-fair vs. fair) × 3 (hemisphere: left vs. medial vs. right) × 3 (region: anterior vs. central vs. posterior) repeated measures ANOVA showed that the main effects of social status and offer fairness failed to reach significance, *p*s > 0.1. The interaction between offer fairness and region was significant, *F*_(4,100)_ = 7.30, *p* < 0.001, *η^2^_partial_* = 0.23. However, further analysis revealed no significant effects of offer fairness in any region (*p*s > 0.1).

### LPP in the 400–700 ms time window on the presentation of UG offers

For ERPs locked with UG offers (Figure 4), in the time window of 400–700 ms, a 2 (social status: high vs. low) × 3 (offer fairness: unfair vs. sub-fair vs. fair) × 3 (hemisphere: left vs. medial vs. right) × 3 (region: anterior vs. central vs. posterior) repeated measures ANOVA revealed a significant main effect of offer fairness, *F*_(2,50)_ = 18.49, *p* < 0.001, *η^2^_partial_* = 0.43, with fair offers eliciting a stronger LPP (2.96 ± 0.42 µV) than unfair offers (2.29 ± 0.40 µV), and unfair offers eliciting a stronger LPP (2.29 ± 0.40 µV) than sub-fair offers (1.66 ± 0.41 µV), *p*s < 0.05. More importantly, the analysis also revealed a marginally significant interaction between offer fairness and social status, *F*_(2,50)_ = 2.86, *p* = 0.067, *η^2^_partial_* = 0.10. Further tests revealed that when participants were in high status, the LPP responses were stronger for both fair offers (2.86 ± 0.51 µV) and unfair offers (2.64 ± 0.46 µV) than for sub-fair offers (1.66 ± 0.41 µV), *p*s < 0.005, with no difference between LPP responses to fair and unfair offers, *p* > 0.1. However, when participants were endowed with low status, the ERP responses were stronger for fair offers (3.07 ± 0.39 µV) than for both unfair (1.94 ± 0.38 µV) and sub-fair offers (1.66 ± 0.48 µV), *p*s < 0.001, with no difference between responses to unfair and sub-fair offers, *p* > 0.1. From a different perspective, while social status did not affect the LPP response to fair and to sub-fair offers, *p*s > 0.1, social status did have a significant effect on unfair offers, *F*_(1,25)_ = 7.68, *p* < 0.05, *η^2^_partial_* = 0.24, with a stronger LPP for the high status condition (2.64 ± 0.46 µV) than for the low status condition (1.94 ± 0.38 µV), *p* < 0.05.

In addition, the interaction between offer fairness and hemisphere was also significant, *F*_(4,100)_ = 7.20, *p* < 0.001, *η^2^_partial_* = 0.22. Tests for simple effects suggested that in the left hemisphere, fair offers (2.71 ± 0.41 µV) elicited a stronger LPP than unfair offers (1.94 ± 0.39 µV), *p* < 0.005, and unfair offers (1.94 ± 0.39 µV) elicited a stronger LPP than sub-fair offers (1.41 ± 0.39 µV), *p* = 0.07; in the medial region, the aforementioned effect of offer fairness remained the same, *p*s < 0.05. However, in the right hemisphere, both fair (2.80 ± 0.35 µV) and unfair offers (2.42 ± 0.35 µV) elicited a stronger LPP than sub-fair offers (1.82 ± 0.34 µV), *p* < 0.001 and *p* < 0.005, respectively, with no difference between LPPs elicited by unfair and fair offers, *p* > 0.1.

## Discussion

In the current study, we used a modified version of UG to investigate whether and how social status influences recipient fairness considerations. Behavioral results revealed that, consistent with previous studies, participant acceptance rates for offers increased with the fairness level of the offers. Moreover, participants were more likely to accept offers when endowed with low status than with high status. Electrophysiologically, the cue indicating low status elicited a more positive P2 than did the cue indicating high status in an earlier time window (170–240 ms); the cue indicating high status elicited a more negative N400 than did the low status cue in a later time window (350–520 ms). For the presentation of UG offers, the LPP in the time window of 400–700 ms was modulated by participants’ social status. Specifically, when the participants were in high status, the LPP for fair and unfair offers was more positive than for sub-fair offers; when the participants were in low status, the LPP for fair offers was more positive than for either sub-fair or unfair offers, which did not differ from each other. Alternatively, while social status modulated LPP responses to unfair offers, with a more positive LPP in the high status than in the low status conditions, it did not modulate LPP to fair and sub-fair offers. Taken together, these findings show that social status can modulate behavioral and neural responses to fairness.

During low status, participants accepted more offers. This finding is consistent with the “entitlement hypothesis” instead of the “emotion hypothesis”: for a given amount of offer, participants were more likely to accept the offer when they expected less than when they expected more. Results in the post-experiment questionnaires showed that participants expected lower offer amounts during low status than during high status, which is consistent with this hypothesis. Although the participants were explicitly instructed that their performance in the time-estimation task had no relation with their final payoffs, the perception of social rank and feelings of entitlement carried over into UG, affecting acceptance rates of unfair and even fair offers from anonymous others. The difference in the feeling of entitlement for individuals in different levels of social status may have arose from a difference in self-esteem. Previous studies showed that obtaining a low rank decreases individuals’ self-esteem (Ellemers et al., 1999) and sense of control over certain situations (Kraus et al., 2009). Compared with individuals in low status, individuals in high status are more concerned with preserving self-esteem (Blader and Chen, 2012). Unfair offers and even some fair offers in the current study were perceived as challenges to one’s self-esteem and rejecting such offers would serve to maintain social standing (Wu et al., 2012).

Although the post-experiment questionnaires also indicated that participants harbored more negative emotions toward unfair offers when they were in low status than in high status, this difference seemed to have no influence on the acceptance rate of UG offers. This finding is obviously inconsistent with other studies showing an increased demand for fairness after negative emotion priming (Harlé and Sanfey, 2007; Grecucci et al., 2013). It is likely that, in the current study, the negative emotion associated with low status was only a by-product and its effect on fairness consideration was overridden by the effect of social status.

### The P2 and N400 effects on social status

The increased P2 amplitudes for cues indicating low status may reflect an enhanced automatic attention to unpleasant stimuli (Carretié et al., 2001, 2004; Gerdes et al., 2013). The P2 is a positive deflection peaking around 200 ms post-onset of the stimuli, and is involved in semantic processing (van Schie et al., 2003), visual feature detection, and selective attention (O’Donnell et al., 1997). Recent studies further suggested that the P2 effect may reflect the evaluation of emotion valence (Schapkin et al., 2000; Wang et al., 2013). For instance, larger P2 amplitudes are found for unpleasant visual stimuli than for pleasant or neutral stimuli, suggesting that the negative valence of emotional stimuli can enhance the early attentional processing of the stimuli (Delplanque et al., 2004; Olofsson and Polich, 2007). In a study on stable and unstable social hierarchies, Zink et al. (2008) found that decreases in social rank led to increased activation in the insula and occipital/parietal cortices, suggesting that a decrease in rank was not only a negative experience, but that it increased participants’ perceptual and attentional processing. Taken as such, we interpret the early increased response to low status information in the current study as a salience marker for critical social information.

The differentiation of low and high status was also present in a later time window (350–550 ms), with a more negative-going N400 for the cue indicating high status than for the cue indicating low status. The enhanced N400 effect for the cue indicating high status may reflect a stronger association between the preexisting representation of the self and positive social information. The N400 is a negative deflection peaking in the period of 300–600 ms post-onset of the stimuli at centro-parietal electrodes. In recent studies, this component was also found to be associated with self-identification (Bentin and Deouell, 2000; Eimer, 2000; Caharel et al., 2006; Butler et al., 2013). For instance, Butler et al. (2013) showed that the N400 amplitude for self and dizygotic twin faces were more negative than for unfamiliar faces. More importantly, when participants viewed self and twin photos over a life span, the N400 only tracked age changes in the self photos, suggesting the N400 as a unique neural response associated with retrieval of stored mental representation of the self in the self-identification process (Butler et al., 2013). According to the theory of self-enhancement, participants are more likely to attend to positive information related to the self (Brown et al., 1988); in the current study, this theory would suggest that participants would be more likely to form positive self-representations by associating themselves more with cues indicating high status than cues indicating low status. The more negative going N400 for high status cues most likely reflects an increased tendency to positively process information related to the self.

### The LPP effects on UG offers

The current study showed a main effect of offer fairness on LPP, with the mean amplitude of LPP being largest for fair offers, intermediate for unfair offers, and smallest for sub-fair offers, which is in line with previous studies (Wu et al., 2011a,b, 2012). These findings may suggest that attentional resources were differentially allocated to the three kinds of offers which had different motivational/arousal significance. The LPP, similar to the P300, is involved in social evaluation (Yeung and Sanfey, 2004; Leng and Zhou, 2010), with increased positive amplitudes reflecting enhanced motivated attention (Hajcak and Olvet, 2008; van Hooff et al., 2011). For instance, the LPP has been reported to be larger for both pleasant and unpleasant pictures than neutral pictures, indicating that more attentional resources are allocated to stimuli that are more motivationally relevant and arousing, irrespective of the emotional valence of the stimuli (Schupp et al., 2003, 2004; Hajcak and Olvet, 2008).

In the present setup, fair offers were linked with the largest reward, sub-fair offers with immediate rewards, and unfair offers with the lowest reward. Certain studies show that P300/LPP tracks reward values, with an enhanced response to a larger reward than a smaller reward (Yeung and Sanfey, 2004; Sato et al., 2005; Leng and Zhou, 2010). If this were the case, in the current study, the amplitude of the LPP should increase with the amount of the offers in UG. On the contrary, we found that LPP amplitudes were larger for both fair and unfair offers than for sub-fair offers. This difference is most likely due to the fact that in previous studies showing that P300/LPP is sensitive to reward magnitude (Yeung and Sanfey, 2004; Leng and Zhou, 2010), the monetary reward was presented as a single number to the participant. Whereas, due to the interactive nature of UG in the current study, the offers not only included the monetary reward but also conveyed social information such as the fairness level and proposer intention. Moreover, previous studies on fairness show that unfair offers are threatening to one’s image of the self (low value, high arousal), whereas fair offers are affirming and abide by social norms (high value, high arousal) (Wu et al., 2011a, 2012). These two kinds of offers are highly likely to have equal or similar motivational/arousal levels and may lead to enhanced motivated attention relative to sub-fair, less salient offers. Therefore, we believe that the LPP effect is modulated by the motivational relevance or arousal intensity corresponding to different fairness levels of the offers, rather than by the reward magnitude of the offers (Hajcak and Olvet, 2008; van Hooff et al., 2011).

Importantly, we found an interaction between social status and offer fairness on the LPP, with different patterns of effects for the high and low status conditions. In the high status condition, LPP responses to fair and unfair offers were both more positive than to sub-fair offers. The lack of difference between LPP responses to fair and unfair offers can be explained from the motivational/arousal perspective outlined above. For individuals in high status, unfair offers are particularly threatening to one’s self-esteem (Wu et al., 2012) and hence are highly arousing, leading to strong LPP responses. For individuals in low status, however, unfair offers were more acceptable and less self-threatening, given that they expected to receive lower offers (i.e., lower minimal acceptable amounts) in this status, as evidenced by the increased acceptance rates of unfair offers while in low status. Thus, the arousal level of unfair offers is closer to the level of sub-fair offers than to the level of fair offers. This explains why LPP was larger for unfair offers in the high status condition than for unfair offers in the low status condition. In addition, social status did not modulate the LPP responses for the sub-fair or fair offers, which may suggest that the motivational/arousal significance of these two types of offers is not affected by social status information.

Two additional points are worth mentioning. First, in this study we did not find an increased MFN for unfair offers over fair offers, inconsistent with other studies using UG (Boksem and De Cremer, 2010; Wu et al., 2011a, 2012; Qu et al., 2013). It is possible that the complexity of the offer structure dampened the earlier MFN responses to fairness. In the current study, participants received twelve different offers (ranging from 1/9–5/5). The diversity of offers was meant to create a more realistic scenario for the participants in UG, but may have rendered the early, automatic computation of fairness less straightforward and resulted in the absence of the fairness effect on MFN. Nevertheless, the system* is* sensitive to fairness in economic decisions, but in a later time window with more advanced processing. Second, to more clearly illustrate the relationship between social status and offer fairness, we chose not to include the middle rank condition in our data analysis. This was because in the current design the opponent’s ranking was not given, making the middle rank susceptible to social comparison (Wu et al., 2011b), which would complicate the effect of status on fairness consideration. In addition, by only analyzing the low and high status, our study took a different position than past studies that have placed their central focus on participants’ perception of higher and lower status others while occupying the middle rank (Zink et al., 2008; Albrecht et al., 2013).

To conclude, by dynamically manipulating the participants’ social rank, this study demonstrated that participants acting as recipients in UG were more likely to accept unfair offers when they acquired a low status compared with a high status. Electrophysiologically, low status information elicited a larger P2 than high status information in an early time window (170–240 ms), while high status information elicited a more negative-going N400 than low status information in a later time window (350–550 ms). These findings suggest that the perception of self-status information involves an earlier automatic attentional processing of the emotional valence of the corresponding social rank and a later working memory process in which the stored representation of the self is retrieved to facilitate self-identification. Additionally, both unfair and fair offers elicited enhanced LPP responses (400–700 ms) when compared with sub-fair offers. Moreover, the LPP was more positive for unfair offers in the high status condition than in the low status condition, suggesting decreased neural salience of unfair offers while occupying a low status position. These findings suggest that the brain responses to fairness in asset division are modulated not only by offer fairness but also by social status, providing further evidence for the context-dependent nature of fairness consideration (Boksem et al., 2012).

## Conflict of interest statement

The authors declare that the research was conducted in the absence of any commercial or financial relationships that could be construed as a potential conflict of interest.
